# Domesticating the beast

**DOI:** 10.1186/1741-7007-11-35

**Published:** 2013-04-15

**Authors:** Virginia Walbot

**Affiliations:** 1 Department of Biology, Stanford University, Stanford, CA 94305-5020, USA

## 

When I wrote my 2009 comment '*Are we training pit bulls to review our manuscripts*?' [1] I was motivated by the aggressive search for flaws that we teach - yes it's our best defense against incomplete scientific evidence for new hypotheses and worse, fraud - but I proposed that we had become overly zealous. Instead of a laser-like focus on demanding more data, more statistics, more of everything, how could we instead teach students to recognize timely, solid, and important findings that actually reflect the type of work most of us publish, most of the time?

The recommendations to give students a better grounding in evaluating typical, very good papers were simple:

1. Read the papers in the bibliographies of 'important' papers and key reviews to learn the context from the past. Note where these papers are published - they are usually in a wide range of journal types.

2. Learn to consider the current state of questions and technologies when a group likely started their work, not what has just become possible to do. Help students see that commitment to an appropriate technology at a specific time is often necessary to get anything done and that jumping to 'the next new thing' may not be possible given the biological materials or the budget.

3. Talk to a group of authors who are writing a current paper about the triage of which data to include, what parts of the manuscript represent confirmatory evidence, and which parts are novel or add a new insight to a current model.

Following the publication of the *Journal of Biology *comment [1], I received nearly 20 emails and many campus comments, and participated in many general discussions at meetings. This is far more feedback than for all but a handful of papers I've written. Several people wanted the list of the 'dirty dozen' papers with fatal flaws to use in their teaching ....hmmmm, did they read the comment? But most people expressed parallel concerns and offered more suggestions on involving students in manuscript writing and in reading the reviews received.

The latter idea is quite good I think, if authors are willing to share this information, because in my experience student evaluations of papers are more thoughtful and thorough than most professional reviews. Students would grasp how some papers with fatal flaws slip through the review and editorial process if they read some of the very superficial reviews authors do receive. Second, students would feel the pain of authors they know when offhand or incorrect comments might doom a manuscript. More importantly, they would see that even enthusiastic reviewers often have excellent suggestions for improving the manuscript, in ways that the authors might not have considered. Many papers are better on rewriting or re-visioning, actually thinking everything through afresh after comments. Students who participated in the rebuttal to reviews would also learn some of the negotiating and 'spin' skills so necessary to advocate for acceptance. Learning to recast the 'state of knowledge' to pinpoint how your report is a step forward often highlights a weakness in the introductory material of the original submission - if reviewers couldn't see how this was new, they are more likely to suggest rejection.

Collectively the active participation of students in manuscript crafting, rewriting, and composing rebuttal letters would prepare them for writing up their own work. I think students would be more realistic about when to start writing, about choosing the appropriate journal for the data in hand (not the dream journal), and how best to anticipate reviewers' comments and write a manuscript that forthrightly addresses legitimate issues, including recognizing that some data types are weaker than others.

One final clarification: I'm a cat person - completely afraid of large dogs, so for me the pit bull inspires terror. On the other hand, quite a few readers own and love this breed, and they were hurt that once again pit bulls were used as a symbol of unnecessary aggression. I apologize and point out that feline hunters show little mercy to their prey; thus, the original article could have been titled are we training tigers or lions or tabbies?

## Note

This article is part of the *BMC Biology *tenth anniversary series. Other articles in this series can be found at http://www.biomedcentral.com/bmcbiol/series/tenthanniversary.
